# Metabolic syndrome in Indian tribes: challenges to reveal its true status

**DOI:** 10.3389/fcdhc.2023.1194471

**Published:** 2023-06-29

**Authors:** Suyesh Shrivastava, Kritika Singh, Tapas Chakma, Arvind Kavishwar

**Affiliations:** National Institute for Research in Tribal Health (ICMR), Jabalpur, India

**Keywords:** metabolic syndrome, hypertension, hyperglycemia, diabetes mellitus, dyslipidemia, obesity, tribes

## Introduction

Metabolic syndrome is a cluster of disorders that increase the risk of cardiovascular diseases (CVD) and Type 2 diabetes. It is the co-occurrence of several known cardiovascular risk factors that are interrelated and contribute to elemental mechanisms and pathways. The key elements of the metabolic syndrome are insulin resistance/hyperglycemia, obesity, dyslipidemia, and hypertension (1). By considering the interrelated factors, the pathophysiology of cardiovascular diseases can be measured. According to various studies (2), metabolic syndrome is dependent on lifestyle and behavioral aspects, which are modifiable risk factors for patients, such as alcohol usage, smoking, physical activity, and dietary patterns – it was observed that modifying these factors can inversely affect the risk of developing metabolic syndrome. The non-modifiable risk factor is represented by genetic components that cannot be modified by lifestyle or dietary changes. Estimating the metabolic syndrome can help identify patients who are at high risk of developing CVD and Type 2 diabetes, in addition to studying the interrelationship between risk factors, which can improve treatment compliance if the disease occurs. Various definitions are proposed for the recognition of the metabolic syndrome.

## Definitions of metabolic syndrome

In 1998, the WHO - World Health Organization (3) was the first to describe and interrelate the key components of the metabolic syndrome, i.e., insulin resistance, obesity, dyslipidemia, and hypertension. To fulfill the WHO definition, insulin resistance (impaired glucose tolerance, impaired fasting glucose, Type 2 diabetes) is mandatory along with other criteria – namely, waist/hip ratio; >0.90 (M), >0.85 (F), BMI >30kg/m^2^, TG ≥ 150 mg/dl, HDL cholesterol: < 35mg/dl (M), <39 mg/dl (F), systolic/diastolic blood pressure ≥140/90 mmHg, and presence of microalbuminuria, i.e., urinary albumin excretion of ≥ 20 µg/min or albumin to creatinine ratio of ≥ 30 mg/g. For the presence of metabolic syndrome, insulin resistance in any form plus two of the other five criteria are mandatory.

In 1999, the European Group for the Study of Insulin Resistance (EGIR) (4) proposed a modified version of the WHO definition. Insulin resistance, described as hyperinsulinemia (i.e., plasma insulin > 75^th^ percentile), is still an absolutely required criterion, but microalbuminuria was eliminated. Other criteria included waist circumference: ≥ 94 cm (M), ≥80 cm (F), TG ≥ 177 mg/dl, HDL cholesterol< 39 mg/dl, systolic/diastolic blood pressure ≥ 140/90 mmHg or Rx. Metabolic syndrome is present if hyperinsulinemia plus any two of the four above-mentioned criteria are present.

In 2003, the American Association of Clinical Endocrinologists (AACE) (5) defined metabolic syndrome as having systolic/diastolic blood pressure ≥ 140/90 mmHg, fasting blood sugar 110-125 mg/dL, and fasting serum triglyceride levels ≥ 150 mg/dL. The AACE criteria are met if the above-mentioned criteria are present, plus there is a family history of Type 2 diabetes, hypertension, or CVD, and a sedentary lifestyle.

The International Diabetes Federation (IDF) in 2005 (6) defined the criteria for metabolic syndrome with central obesity (waist circumference ≥ 94 cm (M), ≥80 cm (F) as mandatory, with geographical variations. Other criteria included fasting glucose ≥ 100 mg/dl, TG ≥ 150 mg/dl or Rx, HDL cholesterol < 40 mg/dl (M), <50 mg/dl (F) or > 130 mmHg systolic or > 85 mmHg diastolic. Metabolic syndrome is present when central obesity plus two of the above four criteria are present.

The National Cholesterol Education Program, Adult Treatment Panel III (NCEP ATP III) (7) revised the criteria in 2000, which were again modified in 2005 by the American Heart Association and the National Heart, Lung, and Blood Institute. According to the NCEP ATP III criteria, metabolic syndrome is diagnosed if three of these five criteria are present: waist circumference >40 inches (M), >35 inches (F), fasting glucose ≥ 100 mg/dl or Rx, HDL cholesterol: <40 mg/dl (M), < 50 mg/dl (F) or Rx, >130 mmHg systolic or > 85 mmHg diastolic or Rx. The NCEP ATP III criteria are the most commonly used, as they do not have any mandatory criteria.

## Metabolic syndrome in tribes

In the present scenario, the lifestyle of tribal populations is transitioning between their authentic way of life and an urban one, which makes them prone to communicable and non-communicable diseases. Various tribes are now exposed to an urban lifestyle that includes the consumption of unhealthy, packaged foods and less of their own, locally produced dishes (8). Additionally, alcohol and tobacco use, which are risk factors for various key elements of the metabolic syndrome, are very common in tribal populations. They are rather susceptible to simultaneous different aspects of the metabolic syndrome. The studies have suggested that hypertension is widespread or even on the rise in tribal populations, with 26% of the individuals there having hypertension (9). As a result of blood glucose levels also being elevated, 1% to 10% of the tribal population is diabetic (10). Studies have also been conducted to determine the prevalence of metabolic syndrome in different Indian tribes, and the results show alarming numbers. A study done on tribal adolescents in Gujrat showed a prevalence of metabolic syndrome of 3.8% (11). Another study correlating metabolic syndrome and cardiovascular disease observed 33.7% and 36.4% metabolic syndrome in the diabetes and prediabetes groups, respectively (12). A different study on two tribes simultaneously also showed a 30% prevalence of metabolic syndrome, including both urban and rural tribes (13).

## Challenges in diagnosing metabolic syndrome in tribal populations

**Figure 1:**
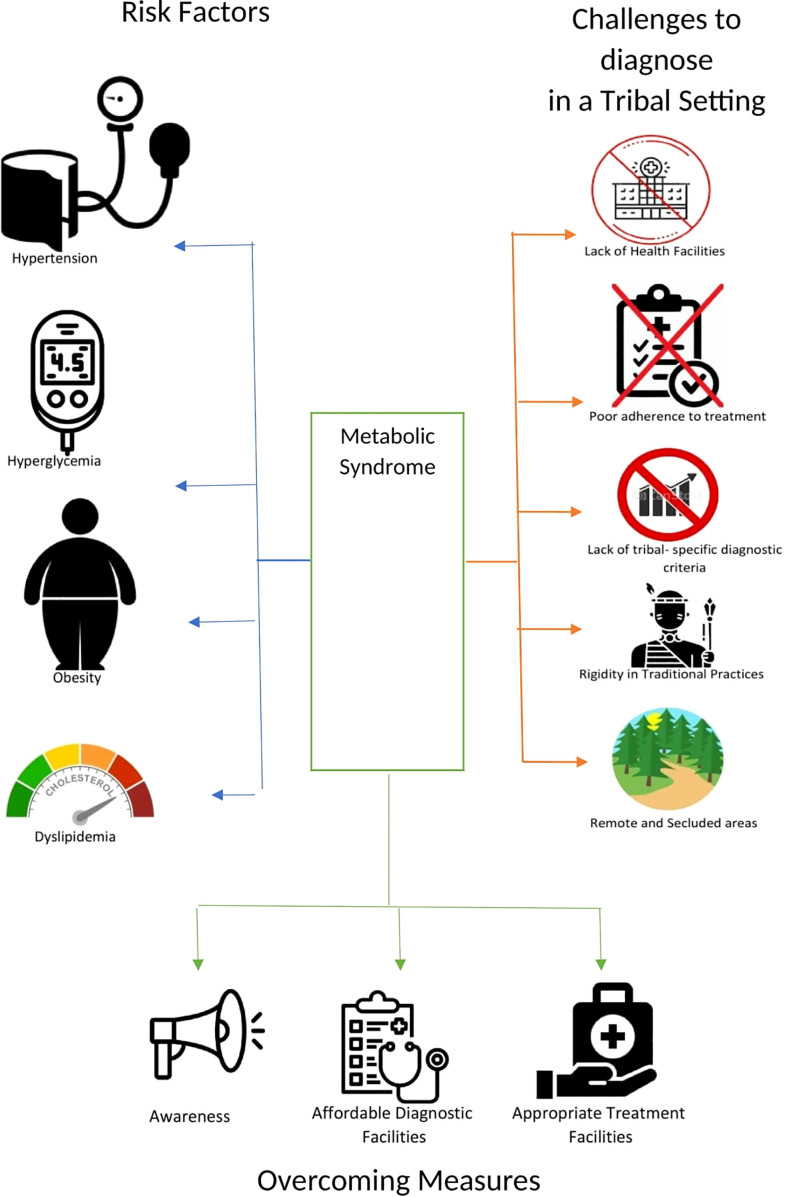
Risk factors for metabolic syndrome, challenges to diagnosis in tribal populations and ways to overcome the challenges.

The study of metabolic syndrome in tribal populations is a paramount task but is more challenging when compared to non-tribal populations (**Figure 1**). The definitions discussed above have mandatory criteria that have to be fulfilled for the recognition of metabolic syndrome. It was often observed that due to malnourishment and nutritional deficiencies, the measurement of waist and hip circumference is less than the normal cut-off value, so even after satisfying the criteria of blood glucose and blood pressure cut-off values, patients do not qualify as having metabolic syndrome, for example, as defined by the IDF. Moreover, the food consumed by tribal populations is based on its availability and not on its nutritional value. In the past, tribal populations used to consume millet, which was rich in micronutrients and had higher nutritional value, but now they are consuming maize and rice more, which are high in carbohydrates and may be responsible for their high blood pressure and blood glucose levels. For the estimation of dyslipidemia, a thorough lipid profile of the respondent is necessary, which requires 2-5 ml of venous blood, and collecting blood samples from tribal populations is a challenging task as they do not have any knowledge or awareness of the procedure. In some cases, it has also been observed that tribes consider providing a blood sample against their belief system. They strongly believe in their traditional healers and their remedies, while having less faith in modern pharmaceuticals. As tribal communities live in remote and secluded areas, sample transportation also becomes a challenging task as there are no laboratories available within a 40-50 km radius, and the only ones that are available are at primary health care centers (PHC) and community health care centers (CHC). Once the sample reaches health centers near the tribal habitats, it may be possible that the whole effort may be useless due to the lack of laboratory facilities for lipid profiles (auto analyzers/semi-automatic biochemistry analyzers), the lack of trained laboratory technicians to perform these tasks, the lack of kits to detect these parameters, or the fact that these kits were not stored at proper temperatures. Lack of awareness and poor adherence to health care in tribal communities are major challenges in understanding the true picture of metabolic syndrome among tribes.

## Clinical challenges of treatment upon diagnosis

As treatment will focus on a) nutrition, b) medication c) mental stress, d) physical activity. The nutritional aspect discussed must include the reintroduction of millet and increased roughage in the diet. Farmers should be encouraged to grow millet and consume it. Some components of antihypertensive/antiglycemics to treat hypertension and diabetes also require a few tests that must be performed before initiation of of therapy, such as treatment with thiazide diuretics or Angiotensin Receptor Blockers (ARBs), patients should be screened for renal failure or hyperuricemia. Similarly, the treatment of hyperlipidemia with atorvastatin (or any HMG-CoA reductase inhibitor) and ezetimibe, as both of these are commonly used drugs, requires monitoring of liver enzymes, which is not possible in peripheral health centers.

Management of diabetes in tribal populations is much more complex than it seems because it comes with challenges like: a) the use of the second-generation sulphonylurea class of drugs requires abstinence from alcohol, which is very difficult; b) the use of biguanide antidiabetic drugs requires screening of the liver and kidney function of the patients before starting them, which is sometimes not possible due to the lack of facilities. Moreover, these drugs cause vitamin B_12_ deficiency; c) the use of α -glucosidase inhibitors causes flatulence and gastrointestinal upset. So, these may lead to poor adherence to treatment; d) the use of insulin, sulphonylurea, and glinides may be associated with hypoglycemic episodes, so these should be used with very close monitoring of the blood glucose levels; e) in terms of prevention, if we think about risk factors, smoking cessation and alcohol abstinence are difficult to achieve in tribal populations.

## Discussion

Metabolic syndrome in tribal populations is becoming a major challenge. First, we need to have a clear picture of the prevalence status among tribes, and only then will the concerned authorities be able to generate the policies that will help to overcome the situation. Knowing the prevalence requires more tribal population-based studies, which have their own challenges. Overcoming these challenges is difficult, but not impossible. The above-mentioned definitions also apply to the urban population, as the IDF definition has obesity as a mandatory criterion, and the WHO and EGIR mandate that insulin resistance is a requirement for diagnosing metabolic syndrome. It is an observed fact that tribal populations deal with malnutrition (14), and if we follow the IDF criteria, a higher number of percentages will not be qualified for the metabolic syndrome positive list. Similarly, it is evident that the urban population has high prevalence of insulin resistance as compared to the tribal communities (15), so if we apply the WHO and EGIR criteria, the results will also not be accurate with respect to the tribal population. The NCEP ATP III criteria can be used to diagnose tribal populations because this definition does not give a preconceived notion of the presence of metabolic syndrome, be it obesity or insulin resistance. If any three out of the five criteria are met, then metabolic syndrome is present, which gives freedom to diagnose the tribal population. Moreover, the lack of awareness about even the basic risk factors of the syndrome in tribal populations is a challenge, so larger-scale awareness campaigns are required to educate indigenous subjects about even the slightest symptoms and risk factors. Children and adolescents should be made aware in schools and educational centers about the ill effects of alcohol and tobacco from an early age, so they are not used in adulthood. There are a lot of challenges to overcome regarding metabolic syndrome in tribal communities, but education, proper diagnosis, and adherence to treatments can positively solve the critical issues described in this study and lead tribal populations to have healthy life.

## Author contributions

SS and KS drafted the manuscript. TC and AK guided and proofread the manuscript. All authors contributed to the article and approved the submitted version.
